# Nicotine pouches, oral cancer and tobacco harm reduction: current evidence and research priorities

**DOI:** 10.3389/froh.2026.1761734

**Published:** 2026-02-09

**Authors:** Giusy Rita Maria La Rosa, Lakshman Perera Samaranayake, Egija Zaura, Iain Chapple, Riccardo Polosa

**Affiliations:** 1 Department of Clinical and Experimental Medicine, University of Catania, Catania, Italy; 2 Faculty of Dentistry, The University Hong Kong, Hong Kong, Hong Kong SAR, China; 3 Global Research Cell, Dr. D. Y. Patil Dental College and Hospital, Dr. D. Y. Patil Vidyapeeth, Pimpri, India; 4 Department of Preventive Dentistry, Academic Centre of Dentistry Amsterdam, Vrije Universiteit Amsterdam and University of Amsterdam, Amsterdam, Netherlands; 5 Periodontal Research Group and Birmingham’s NIHR Biomedical Research Center in Inflammation, University of Birmingham, Birmingham, United Kingdom; 6 Center for the Acceleration of Harm Reduction, University of Catania, Catania, Italy

**Keywords:** nicotine pouches, oral cancer, oral health, smokeless tobacco, smoking

## Abstract

Tobacco smoking remains the most consistent and preventable risk factor for oral cancer, driven by exposure to combustion-derived toxins that promote DNA damage, inflammation and microbiota dysregulation. Global data show substantial geographic variability in disease burden, with particularly high incidence and mortality especially in South and Southeast Asia, where culturally reinforced and deeply embedded forms of high-nitrosamine smokeless tobacco and areca nut continue to drive risk. In this evolving landscape, nicotine pouches have rapidly expanded as tobacco-free oral products manufactured to deliver nicotine without combustion. Toxicological analyses reveal significantly lower levels of harmful constituents relative to cigarettes and traditional smokeless tobacco, and short-term clinical studies report reductions in oral mucosal irritation and gingival inflammation among exclusive users. However, no long-term epidemiological evidence is currently available to assess their potential impact upon oral carcinogenesis, and existing human studies remain few, small and heterogeneous. This mini review highlights critical priorities for research, including the need for long-term prospective studies, standardized product testing, independent toxicological assessments and surveillance of patterns of use, dual use and youth uptake. The integration of harm reduction approaches with established prevention strategies may offer opportunities to mitigate oral cancer risk in adults who smoke and/or consume unregulated smokeless tobacco products with high risk profiles that are very common in Asia, the Middle East and Africa. However, this approach requires cautious interpretation of the current evidence and ongoing monitoring of emerging products.

## 1 Introduction

Oral cancer, most commonly presenting as oral squamous cell carcinoma (OSCC), is the thirteenth most common malignancy worldwide and represents a significant public health challenge due to its high morbidity, frequent late diagnosis and the substantial functional and psychosocial consequences of treatment (1–3). Moreover, global projections indicate that the broader group of head and neck cancers will rise by nearly 30 percent by 2030 (4), underscoring the need for strengthened prevention strategies. The etiology of oral cancer is multifactorial and includes well established determinants such as tobacco use, harmful alcohol consumption, chronic mucosal irritation and infection with high-risk human papillomavirus (HPV) (5–7). Among these, tobacco smoking remains the most consistent and preventable risk factor across populations.

**Table 1:** General characteristics of the studies investigating oral health effects associated with nicotine pouch use.

Author, Year	Country	Study design	Sample size	Follow-up (weeks)	Comparison	Outcome	Main finding
Alizadehgharib, 2022 (45)	Sweden	Prospective single cohort	60	6	Within subject	Oral mucosa lesions	A 70% reduction of pre-existing snus-associated lesions was reported.
						Gingival retraction	Gingival retraction varied between 54% and 57%, with no change during the study.
La Rosa, 2025 (47)	Sweden	Prospective single cohort	23	5	Within subject	Oral mucosa lesions	Snus lesion prevalence declined from 95.7% (*n* = 22) to 69.6% (*n* = 16), with lesion severity significantly reduced.
						Gingival irritation/gingivitis	Gingival irritation decreased by 90.0%; self-reported gingivitis cases (*n* = 3) were eliminated.
						Gingival retraction	Gingival retraction was 39.1% (*n* = 9), with no change during the study.
Liu, 2025 (46)	USA	Randomized, open-label, parallel-group	149	24	Smokers	Gingival inflammation/bleeding (MGI, BI, PPD, BOP, GCF)	Nicotine pouch users MGI and BI showed statistically significant reductions at week 12 (MGI 20%; BI 30%) and week 24 (MGI 28%; BI 23%) compared with smokers and baseline. No significant changes for PPD and BOP. Significant reductions from the baseline in mean GCF volume were shown (−17.3 µL) at week 12 and (−20 µL) at week 24.
						Plaque (TPI)	No significant changes.
						Stains (LSI)	Statistically significant reductions (∼60%) were reported for NP users vs. smokers.
Miluna-Meldere, 2024 (48)	Latvia	Case series	5	–	Within subject	Oral mucosa lesions	White mucosal lesions were observed at pouch placement sites, with histopathology showing parakeratosis, acanthosis, oedema and chronic inflammatory infiltration, indicating measurable cellular level changes associated with pouch use.

BI, Gingival Bleeding Index; BOP, Bleeding on Probing; GCF, Gingival Crevicular Fluid; LSI, Lobene Stain Index; MGI, Modified Gingival Index; NP, nicotine pouch; TPI, Turesky Plaque Index; PPD, Periodontal Probing Depth.

Over the past decade, the nicotine use landscape has diversified, with the introduction of non-combustible products aimed at reducing exposure to toxins produced by smoking (8). Nicotine pouches have emerged as one of the most rapidly expanding categories within this portfolio of harm reduction strategies (9, 10). In several geographic regions, including Asia, the Middle East, and Africa, the widespread use of regional smokeless tobacco products represents a major public health concern and contributes substantially to the burden of oral cancer (11).

The purpose of this mini review is to examine how nicotine pouches and other alternative nicotine products align with current knowledge on smoking related oral cancer risk and to discuss their potential role within broader tobacco harm reduction strategies.

## 2 Methods

A search of PubMed and Google Scholar was conducted using key terms including “oral cancer”, “smoking”, “oral health”, “smokeless tobacco”, and “nicotine pouches”. Priority was given to recent peer reviewed articles and global reports for epidemiological and clinical data on oral cancer, and to human clinical studies on nicotine pouches, in order to identify, without restrictions on study design, studies that examined the oral health effects of exclusive oral nicotine pouch use. The aim was to collate and synthesize current evidence on the established link between smoking and oral cancer and to discuss how emerging non-combustible nicotine products such as nicotine pouches should be evaluated within this broader risk context, while outlining key priorities for future research.

## 3 Smoking as a primary modifiable risk factor for oral cancer

The role of tobacco smoking in the development of oral cancer is supported by extensive epidemiological, experimental and mechanistic evidence (12–14). Cigarettes contain over 7,000 chemicals, many of which are toxic and at least 69 are known to cause cancer, including polycyclic aromatic hydrocarbons, volatile aldehydes and tobacco specific nitrosamines (15, 16). These combustion-derived compounds promote mutations, DNA adduct formation and chronic inflammatory processes that facilitate malignant transformation in the oral cavity, mediated also by alterations in the oral microbiota (17, 18). The combination of smoking and alcohol consumption exerts a synergistic effect, producing risk levels far higher than those associated with either exposure alone. A recent meta-analysis quantified this interaction, reporting that heavy alcohol use combined with heavy smoking increases the risk of oral cancer by more than thirtyfold (RR: 36.42, 95% CI: 24.62–53.87) (19).

**Figure 1:**
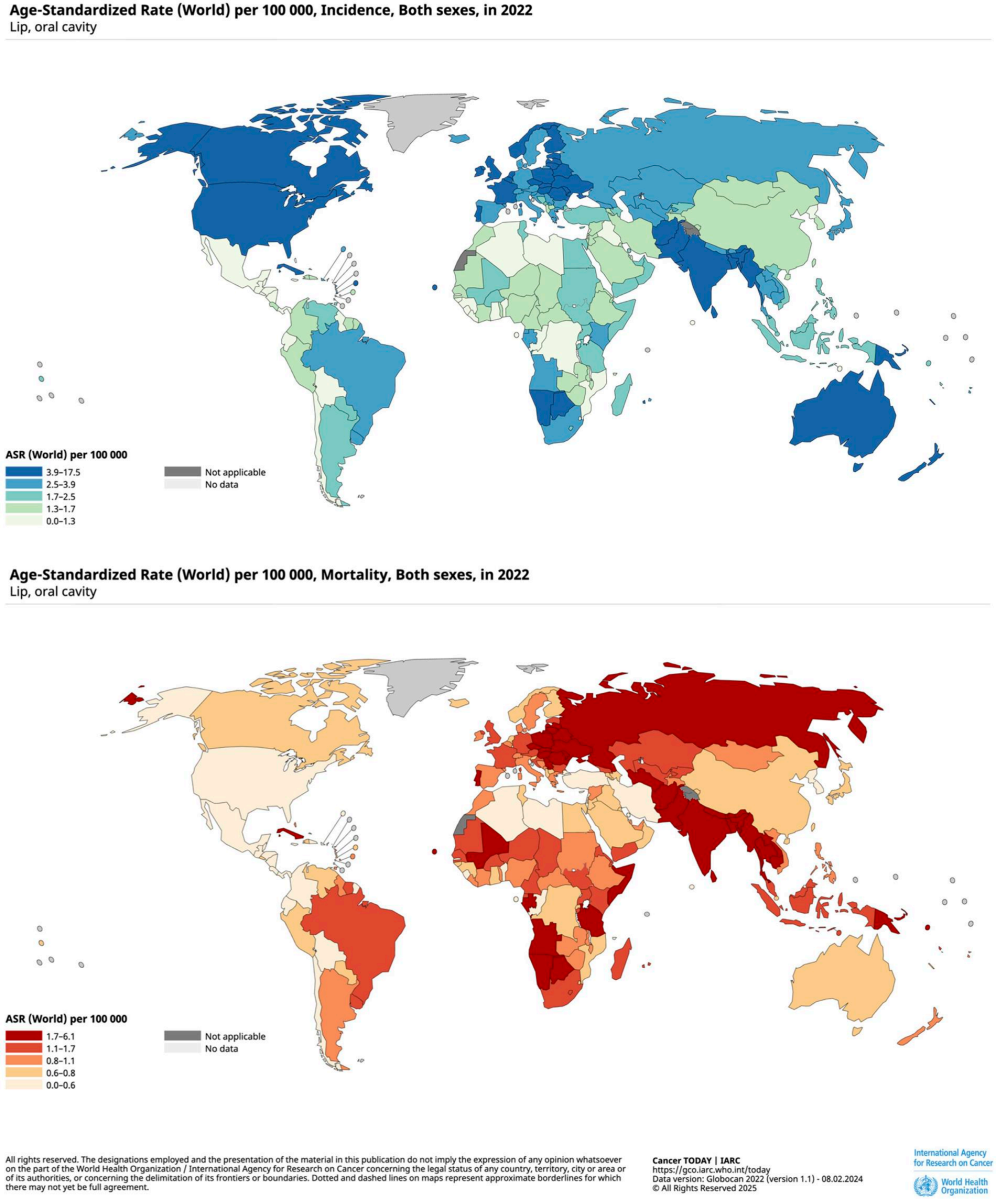
Age-standardized incidence and mortality rates of lip and oral cavity cancer per 100,000 population in the world, both sexes, in 2022. Reprinted from Cancer TODAY | IARC. Available at: https://gco.iarc.who.int/today. Data version: Globocan 2022 (version 1.1)—08.02.2024 (Accessed November 25, 2025).

Globally, the burden of oral cancer is unevenly distributed. High incidence and mortality rates are observed in South and Southeast Asia, parts of the Western Pacific and several regions of Europe (20) (Figure 1). These geographical differences reflect variations in behavioral risk factors and cultural practices. In several countries, specific forms of smokeless tobacco use characterized by high concentrations of carcinogens continue to be widely consumed, often involving products that are not authorized or regulated (21). The high persistence is explained in part by deep-rooted cultural practices involving traditional forms of tobacco use, by regulatory policies that are inconsistently enforced, together with limited access to cessation services (22–24). The cumulative effect of long-term exposure to such agents, combined with pronounced socioeconomic inequalities, continues to sustain a substantial underlying population risk.

This broader epidemiological framework provides the foundation for evaluating how harm reduction strategies could complement conventional approaches to prevention and contribute to limiting the projected burden of oral cancer in the next decade and beyond.

### 3.1 The unique burden of oral cancer in south Asia: smokeless tobacco

South and Southeast Asia, which report the highest incidence and mortality rates for this disease, represent some of the strongest examples of how cultural practices and product types shape oral cancer risk (25). In countries such as India, Bangladesh, Pakistan and Sri Lanka, the use of smokeless tobacco is deeply embedded in society through social traditions. Products such as gutkha, zarda, khaini, naswar and paan combined with tobacco are widely consumed across age groups and socioeconomic backgrounds (26–28). Areca nut, consumed by an estimated 600 million people worldwide, is the seed of the Areca catechu palm and is typically chewed alongside betel quid. This preparation traditionally combines sliced areca nut, slaked lime and betel leaf, and is often supplemented with tobacco, sweeteners or flavoring agents (29).

Tobacco-specific nitrosamines (TSNAs), particularly N′-nitrosonornicotine (NNN) and 4-(methylnitrosamino)-1-(3-pyridyl)-1-butanone (NNK), which are present at high concentrations in most smokeless tobacco products, are classified as carcinogenic to humans (Group 1) by the International Agency for Research on Cancer (28). Their carcinogenic activity is mediated through the formation of DNA adducts and mutations, as well as through receptor-mediated pathways that promote tumor development and progression (12, 30). Evidence from experimental rodent models suggests a direct association between exposure to NNN and NNK and the induction of oral tumors, supporting their biological plausibility as key drivers of oral cancer risk (31, 32). In addition, the mechanical friction generated by chewing and the chemical irritants contained in areca nut contribute to chronic mucosal trauma and facilitate the development of pathological changes (33).

Epidemiological studies consistently demonstrate strong associations between smokeless tobacco use and oral cancer (34, 35). Habitual use of areca nut and betel quid is strongly associated with the onset of oral potentially malignant disorders such as oral submucous fibrosis and plays a central role in the development of oral squamous cell carcinoma (36, 37). A recent systematic review reported a strong association between the use of tobacco-containing betel quid and oral cancer incidence, with an odds ratio of 7.74 (95% CI: 5.38–11.13) (35).

Taken together, these findings illustrate how traditional smokeless tobacco and areca nut practices continue to drive a substantial share of the global oral cancer burden. Within this evolving landscape, attention has increasingly shifted toward alternative nicotine products that differ markedly from conventional smokeless forms of tobacco.

### 3.2 The case of Swedish snus: epidemiological evidence

The case of Swedish snus has received particular attention in epidemiological research due to its substantially lower toxicant profile compared with conventional smokeless tobacco products. This reduced toxicant burden is largely attributable to the pasteurization process used in snus manufacturing, which results in markedly lower levels of TSNAs, estimated to be approximately 73% lower than those found in traditional oral tobacco products marketed in the United States (38). Although snus is not risk-free, its use has been associated with a substantial reduction in harm when employed as a substitute for combustible tobacco (39). In Sweden, where snus has largely replaced cigarette smoking among men, tobacco-related mortality rates are among the lowest in Europe (40). With a specific focus on oral cancer risk, a pooled analysis including more than 418,000 Swedish men found no significant association between snus use and oral cancer incidence (adjusted hazard ratio: 0.90, 95% confidence interval: 0.74–1.09) (41). These findings have been further corroborated by a recent systematic review, which concluded that snus use does not appear to be associated with an increased risk of oral cancer (42).

## 4 Transitions in the nicotine market: the emergence of nicotine pouches

In the last decade, the nicotine product market has expanded to include several non-combustible products designed to deliver nicotine without the toxins generated by burning tobacco (43). Among these, nicotine pouches have emerged as one of the most dynamic categories. These products are small oral sachets containing nicotine, plant-based fillers, flavorings and food grade additives. They do not contain tobacco leaf and do not require combustion or spitting (44–46). Whilst their mode of use resembles that of traditional snus, they differ substantially in composition (tobacco-free), manufacturing processes and regulatory classification (47, 48).

The rapid growth of nicotine pouches is driven by multiple factors, including discreet use, a wide range of flavors, relatively low cost and the perception of lower risk compared with cigarettes (49, 50). From a toxicological perspective, although nicotine pouches are not risk free, chemical analyses consistently show that they contain significantly lower levels of harmful constituents than both traditional cigarettes and classical snus, which in itself already contains fewer toxins than combusted tobacco (48, 51). Existing evidence indicates that tobacco free nicotine pouches are generally well tolerated, with oral adverse events mainly limited to mild soreness of the mouth, dry mouth or transient mucosal irritation. In January 2025, the FDA authorized the marketing of 10 oral nicotine pouch variants, concluding that their use would be “appropriate for the protection of public health” (52). Their decision was based on toxicological data, usage patterns, and population-level impact data.

Some studies suggest that nicotine pouches may contribute to a reduction in cigarette consumption compared with control conditions, with effects comparable to those seen with snus or nicotine gum. However, current data do not demonstrate statistically significant improvements in smoking cessation rates relative to other nicotine products or control groups (53).

Although the toxin profile of nicotine pouches is clearly more favorable than that of cigarettes and traditional smokeless products, this represents only one dimension of a broader public health debate. Patterns of use, long term outcomes, youth uptake and the potential implications for health effects, particularly oral cancer prevention remain critical considerations requiring careful evaluation (54).

### 4.1 Oral health impact of nicotine pouches and oral cancer risk: what is known

Current evidence on the oral health impact of nicotine pouches remains limited but is progressively increasing. To date, only four studies have investigated their effects on oral tissues, adopting heterogeneous study designs and varying follow-up durations (55–58) (Table 1). Two of these prospective within-subject studies assessed changes in oral mucosa amongst current users, consistently reporting a reduction in pre-existing snus-associated lesions or an improvement in lesion severity over short follow-up periods (55, 57). A single randomized controlled trial compared the effects of switching from cigarettes to nicotine pouches, demonstrating statistically significant reductions in gingival inflammation, with improvements in Modified Gingival Index (MGI) and Gingival Bleeding Index (BI) at 12 and 24 weeks, while periodontal probing depth and bleeding on probing remained unchanged (56).

Given the limited number of available studies, the lack of a control group in almost all current studies, the absence of long-term prospective data, and the relatively recent introduction of these products, the long-term implications of nicotine pouches in oral carcinogenesis remain unclear. Existing research has primarily focused on mucosal changes related to prior snus use, reporting improvements in lesion prevalence or severity, yet these findings are based on small samples, limited follow-up periods and self-reported outcomes, which restrict the strength of evidence (55, 57). Because oral cancer develops over long latency periods, products that have been on the market for less than a decade cannot yet be fully evaluated via epidemiological approaches applied to cigarettes or traditional smokeless tobacco. In addition, the heterogeneity of products available on the market complicates the extrapolation of results from one brand or formulation to another. This underscores the need for standardized testing protocols and transparent reporting of product constituents.

Nevertheless, toxicological assessments indicate that switching completely from smoking to nicotine pouches may substantially reduce exposure to established carcinogens (51, 59). Policymakers and public health professionals must carefully balance this potential with the need to avoid unintended consequences such as dual use, insufficient product regulation and youth uptake. Advancing the evidence base will require long-term randomized controlled trials and large-scale longitudinal observational studies capable of evaluating chronic exposure, mucosal changes and potential cancer-related endpoints, thereby supporting regulatory and clinical decision making with robust and transparent data.

## 5 Future directions: surveillance, research and integrated harm reduction policies

To fully assess the potential of nicotine pouches in reducing oral cancer risk compared with current smoking, several lines of research must be strengthened. First, longitudinal studies are needed to evaluate temporal mucosal changes over a substantive period, the incidence of premalignant conditions and long-term carcinogenic outcomes. Second, comparative studies should examine nicotine pouch use alongside smoking, snus, heated tobacco and traditional smokeless products to contextualize relative risk.

A recent network meta-analysis assessing oral health effects across non-combustible nicotine products did not identify any eligible randomized controlled trials on nicotine pouches, and the relatively short follow up period of the included studies did not allow for evaluation of their potential impact on oral carcinogenesis (60, 61). Although RCTs are required to determine the effectiveness or safety of nicotine products as smoking cessation tools, outcomes related to latency, such as oral cancer, require long-term observational studies. Such studies are critical to generate evidence on chronic exposure and to assess the real world health impact of these products over extended periods. Furthermore, toxicological research should continue to monitor nitrosamine levels and novel carcinogens in new formulations. Of note, most of the available evidence on toxicological profiles comes from industry funded studies (51, 59, 62), underscoring the need for independent, long term research to validate current findings and inform evidence-based regulatory decisions.

Epidemiological surveillance is equally critical. Countries with rising nicotine pouch use need to monitor user profiles, transitions from smoking, dual use patterns and youth access. Regulatory frameworks should promote quality standards, child resistant packaging, responsible marketing practices and transparent labeling. Public health communication must clearly differentiate the relative risk levels of available nicotine products, ensuring accurate and balanced information that neither minimizes documented harms nor overstates potential risks.

A comprehensive strategy for oral cancer prevention should integrate three interrelated components. First, reducing the initiation of all tobacco products as a primordial prevention approach. Second, facilitating complete cessation as a primary prevention strategy; and finally providing safer alternatives for adults who cannot or will not quit smoking. However, their integration into harm reduction strategies must be accompanied by continuous surveillance, transparent reporting, and rigorous long-term research to evaluate safety, usage patterns, and population-level health outcomes.

Given the current state of the literature, this review was intentionally framed as exploratory. The available evidence remains limited and heterogeneous, precluding definitive conclusions regarding clinical applications. Accordingly, the primary focus of this work is to provide an organized overview of existing studies and to critically discuss their findings within the broader context of oral cancer research. By mapping the current evidence and highlighting methodological constraints and unresolved questions, this review seeks to inform ongoing scholarly debate and to identify priorities for future high quality primary research, which is necessary before more conclusive interpretations can be supported.

## 6 Conclusions

The shift from combustible tobacco use to that of low toxin-containing nicotine products presents both opportunities and challenges for reducing oral cancer risk among adults who smoke. Smoking remains the primary cause of oral cancer globally, and the burden of disease is particularly pronounced in regions where high nitrosamine smokeless products are widely used. By eliminating combustion and the use of tobacco leaf, nicotine pouches have a substantially lower toxin profile and may reduce exposure to carcinogens in individuals who switch completely from smoking. However, the absence of long-term evidence requires a cautious and balanced interpretation of the potential for these products to present a lower risk compared to combustible tobacco. Sustained research efforts, continuous epidemiological surveillance and responsible regulatory oversight will be essential to determine the role that nicotine pouches may play in strategies designed to lower oral cancer risk in smokers and support long term smoking cessation.
